# Concurrent Bladder Urothelial Carcinoma and Prostate Tuberculosis: A Case Report

**DOI:** 10.1002/iju5.70195

**Published:** 2026-05-15

**Authors:** Rikuo Suzuki, Taketo Kawai

**Affiliations:** ^1^ Department of Urology International University of Health and Welfare Ichikawa Hospital Chiba Japan; ^2^ Department of Urology National Center for Global Health and Medicine Tokyo Japan

**Keywords:** bladder cancer, prostate tuberculosis, pulmonary tuberculosis, urogenital tuberculosis, urothelial carcinoma

## Abstract

**Introduction:**

Tuberculosis is associated with an increased risk of bladder cancer; however, the simultaneous occurrence of urogenital tuberculosis and bladder cancer is rare. Primary prostate tuberculosis is relatively uncommon in patients with urogenital tuberculosis.

**Case Presentation:**

A 68‐year‐old man presented with gross hematuria and irritative voiding symptoms. Cystoscopy revealed a bladder tumor. The PSA level was elevated (8.32 ng/mL), and MRI findings suggested prostate cancer. Chest radiography and CT indicated pulmonary tuberculosis, which was confirmed by sputum mycobacterial culture and PCR. Urine mycobacterial cultures revealed urinary tuberculosis. TURBT and prostate biopsy were performed after initiating treatment for tuberculosis and confirming sputum conversion. Histopathological examination revealed urothelial carcinoma of the bladder and prostate tuberculosis.

**Conclusion:**

This is the first reported case of concurrent bladder urothelial carcinoma and prostate tuberculosis. Patients with pulmonary tuberculosis may benefit from evaluation for urogenital tuberculosis, including prostatic involvement, and consideration of coexisting urothelial carcinoma.

## Keynote Message

This is the first documented case of concurrent bladder urothelial carcinoma and prostate tuberculosis. This case emphasizes that patients with pulmonary tuberculosis may have unrecognized urogenital involvement, including prostatic diseases. This also highlights the fact that patients with urogenital tuberculosis are at an elevated risk of developing urothelial carcinoma. Persistent sterile pyuria or atypical urinary findings should prompt clinicians to consider tuberculosis in differential diagnoses. Early recognition of coexisting tuberculosis and malignancy is essential for timely and appropriate management.

**Abbreviations**
ADC: apparent diffusion coefficient
BCG: Bacillus Calmette–Guérin
CT: computed tomography
DWI: diffusion‐weighted image
HbA1c: hemoglobin A1c
MRI: magnetic resonance imaging
PCR: polymerase chain reaction
PI‐RADS: Prostate Imaging Reporting and Data System
PSA: prostate specific antigen
T2WI: T2‐weighted image
TB: tuberculosis
TURBT: transurethral resection of bladder tumor
UGTB: urogenital tuberculosis

## 1 Introduction

Tuberculosis (TB) was once a devastating infectious disease worldwide; however, its incidence has markedly declined with advances in public health, preventive medicine, and pharmacological therapy, particularly in developed countries. In Japan, the incidence reached the threshold for a low‐burden country in 2021 and was 8.1 per 100 000 population in 2024 [1]. Urogenital tuberculosis (UGTB) is the second most common form of extrapulmonary TB after lymph node involvement, accounting for 15%–20% of pulmonary TB cases in developing countries but only 2%–10% in developed countries [2]. Herein, we report a rare case of concurrent bladder cancer and prostate TB.

## 2 Case Presentation

**FIGURE 1:**
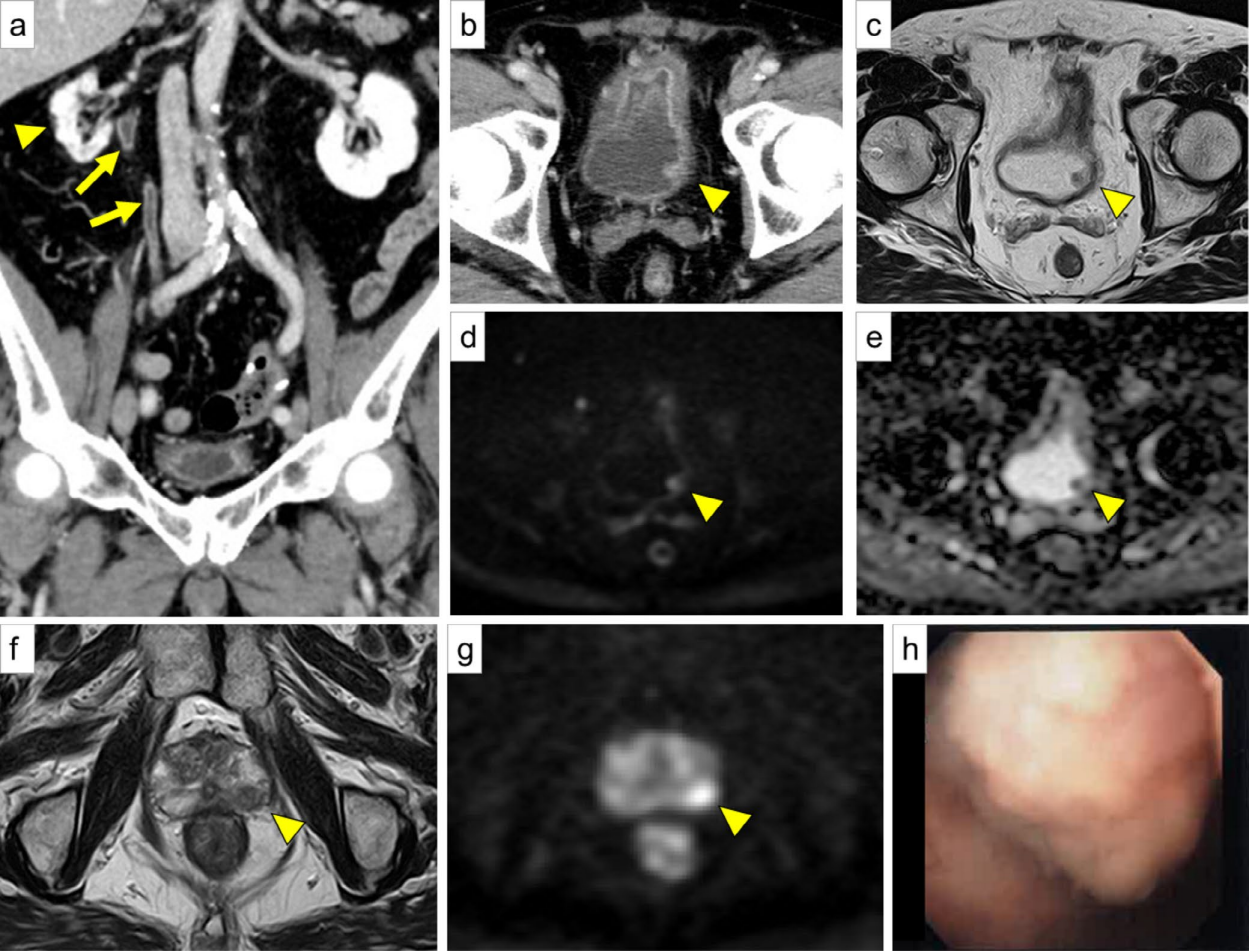
Imaging and cystoscopic findings at initial presentation. (a, b) Contrast‐enhanced abdominal CT; (a) right renal atrophy (arrowhead) and right ureteral wall thickening (arrow). (b) Suspected tumor on the left lateral bladder wall (arrowhead). (c–e) Bladder MRI; (c) T2WI, (d) DWI, and (e) ADC map. Suspected non–muscle‐invasive clinical T1 bladder cancer (arrowhead). (f, g) Prostate MRI; (f) T2WI and (g) DWI. A PI‐RADS v2.1 category 4 lesion suspicious for prostate cancer in the dorsal peripheral zone of the left lobe (arrowhead). (h) Cystoscopy showing a non‐papillary mass on the left lateral bladder wall (limited by cloudy urine).

A 68‐year‐old man presented with frequent urination and incomplete emptying. Pyuria was detected and levofloxacin was prescribed. He later developed gross hematuria and was referred to our hospital. Urinalysis revealed pyuria; however, urine culture and cytology were negative. Blood tests revealed newly diagnosed diabetes mellitus (HbA1c 8.6%) and elevated PSA (8.32 ng/mL). CT revealed right renal atrophy, ureteral wall thickening, and a suspected tumor (Figure 1a,b). MRI suggested non–muscle‐invasive, clinical T1 bladder cancer (Figure 1c–e) and a PI‐RADS v2.1 category 4 lesion suspicious for prostate cancer (Figure 1f,g). Although limited by cloudy urine, cystoscopy revealed a non‐papillary mass on the left lateral bladder wall (Figure 1h).

**FIGURE 2:**
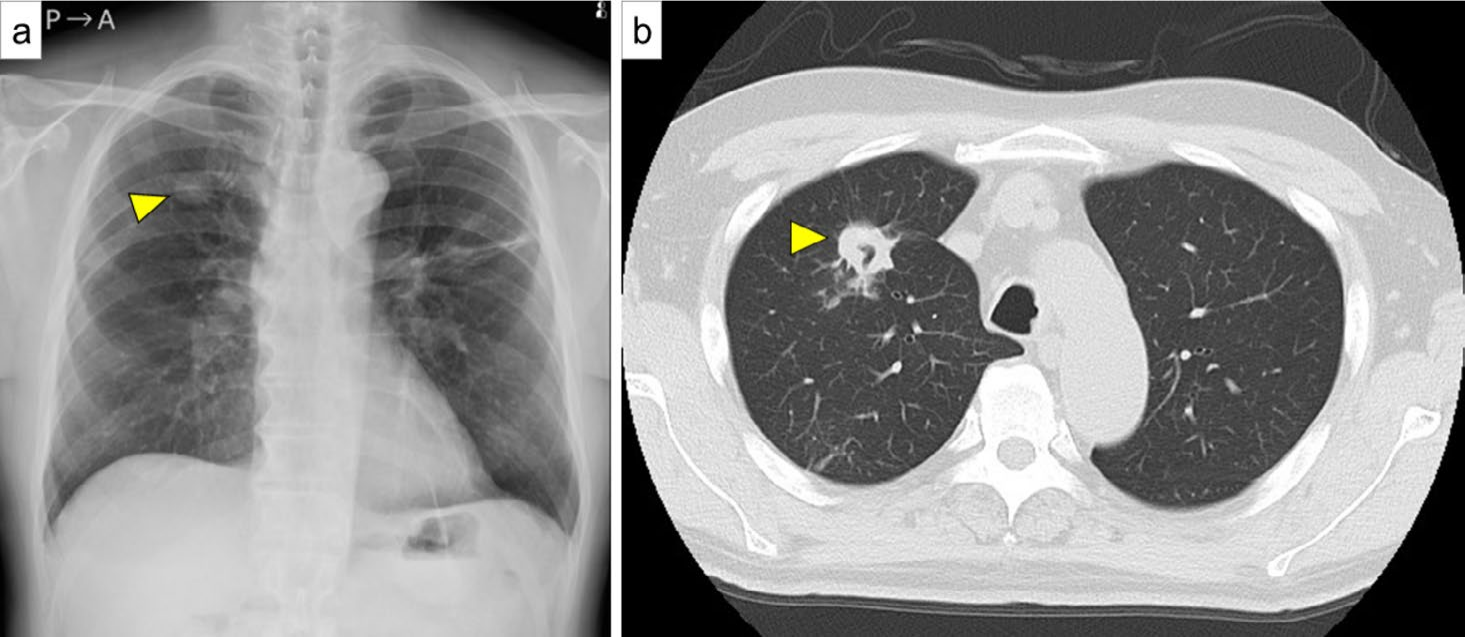
Chest imaging findings. (a) X‐ray showing a mass in the right upper lung field (arrowhead). (b) CT demonstrating a cavitary lesion (arrowhead).

TURBT, ureteroscopy, and prostate biopsy were planned and glycemic control was initiated. However, preoperative chest radiography revealed a right upper lung nodule (Figure 2a), and CT revealed a cavitary lesion (Figure 2b). Sputum mycobacterial culture and PCR were positive for *Mycobacterium tuberculosis* , leading to the diagnosis of active pulmonary TB. Furthermore, *M. tuberculosis* was detected in urine mycobacterial cultures, raising the possibility that the urinary and prostatic lesions were caused by TB‐related inflammatory changes. Antituberculous therapy with rifampicin, isoniazid, and ethambutol was initiated, and sputum and urine mycobacterial culture conversions were confirmed after 2 months.

**FIGURE 3:**
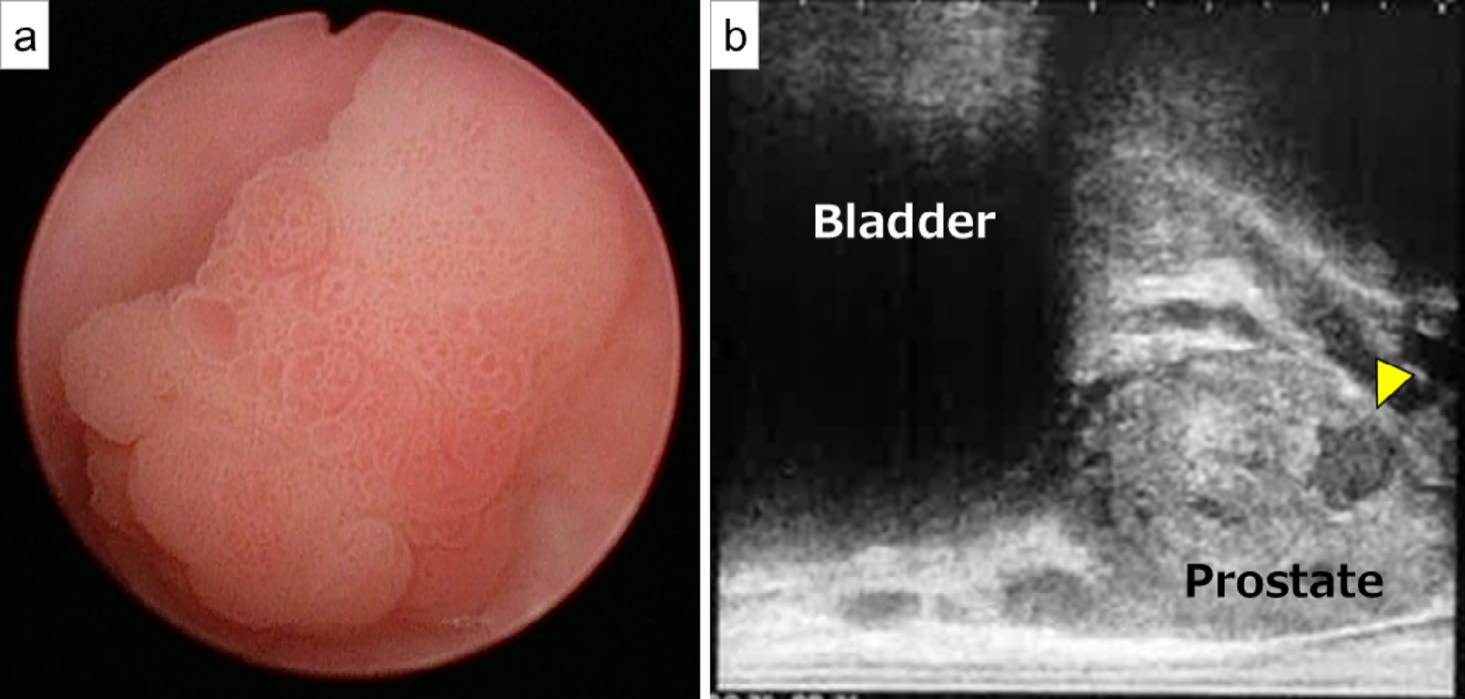
Intraoperative findings. (a) Cystoscopy showing a papillary tumor on the left lateral bladder wall. (b) Transrectal ultrasonography showing a hypoechoic mass within the prostate (arrowhead).

**FIGURE 4:**
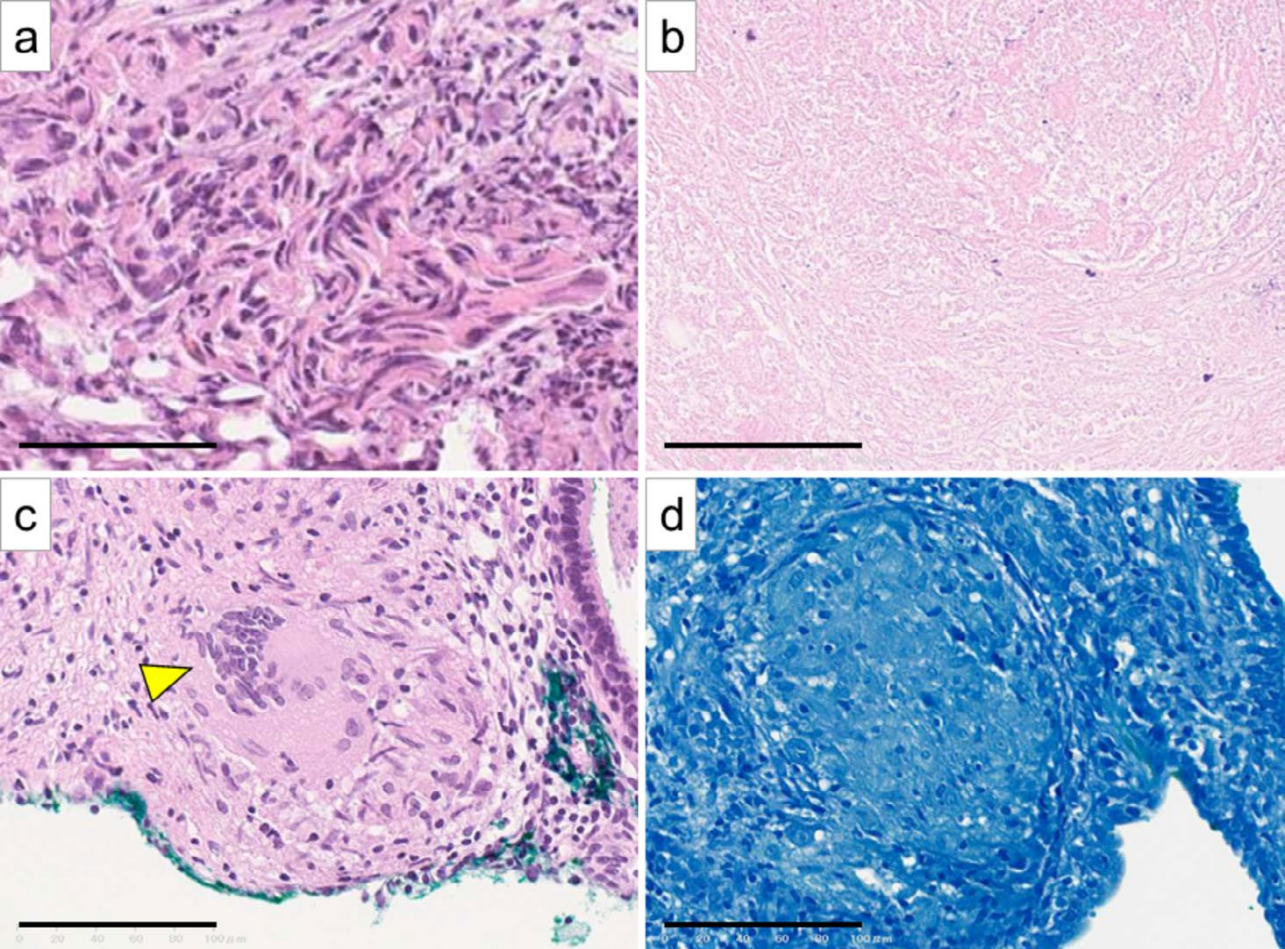
Histopathological findings. (a) TURBT specimen, hematoxylin and eosin staining; high‐grade urothelial carcinoma, G3, pT1. (b–d) Prostate biopsy specimens. (b, c) Hematoxylin and eosin staining; (b) caseous necrosis, and (c) Langhans giant cells (arrowhead). (d) Ziehl–Neelsen staining; negative. All scale bars indicate 100 μm.

Despite antituberculous treatment, the bladder mass persisted and PSA remained elevated at 7.72 ng/mL. TURBT and prostate biopsy were performed. Intraoperatively, the bladder tumor appeared papillary, possibly due to the resolution of pyuria (Figure 3a), and transrectal ultrasonography revealed multiple hypoechoic lesions within the prostate (Figure 3b). Histopathology of the TURBT showed high‐grade urothelial carcinoma, G3, pT1 (Figure 4a). Prostate biopsy showed caseous necrosis and Langhans giant cells without malignancy, consistent with prostate TB, although Ziehl–Neelsen staining was negative (Figure 4b–d).

A second TUR was performed 1 month later, which showed no residual tumor. Ureteroscopy revealed no ureteral tumor, and biopsy of the coarsely irregular mucosa revealed no malignancy or TB. A review of the CT scan performed at another hospital 3 years earlier identified a 10‐mm right ureteral stone, likely explaining the right renal atrophy and ureteral inflammatory changes.

Because antituberculous agents are considered to antagonize intravesical BCG therapy [3], intravesical BCG was deemed inappropriate during ongoing antituberculous treatment and was therefore not administered. As no residual tumor was detected at the second TUR, careful cystoscopic surveillance was planned for the follow‐up. For prostate TB, PSA monitoring and MRI follow‐up will be performed to assess treatment response. At present, 6 months have elapsed since the second TUR, and no recurrence of bladder cancer or elevation in PSA has been observed.

## 3 Discussion

BCG immunotherapy for bladder cancer was developed based on its immune‐activating antitumor effects [4], raising the question of whether TB confers protection against bladder cancer. However, recent evidence has not supported this hypothesis. A meta‐analysis found that individuals with prior TB had an increased risk of several cancers, including an approximately twofold higher risk of bladder cancer [5]. One explanation is that occult malignancies may compromise host immunity, predisposing individuals to TB activation. Furthermore, patients with prior UGTB have a higher incidence of urothelial carcinoma than those with non‐urogenital TB [6], suggesting that chronic inflammation associated with TB may contribute to carcinogenesis. Although synchronous urothelial carcinoma with renal or bladder TB has been reported [7, 8, 9, 10], no previous report has described synchronous prostate TB. A PubMed search (keywords: “bladder urothelial carcinoma” and “prostate tuberculosis,” up to March 2026) identified no relevant reports.

Prostate TB commonly coexists with renal or other UGTB, whereas isolated cases represent only 5%–7% of UGTB cases [11, 12, 13]. The presumed routes of infection include hematogenous spread or descending infection from the urinary tract [12, 13, 14]. Clinical symptoms are generally nonspecific, such as irritative voiding symptoms [12, 13, 14]. Transient elevations in PSA levels have been described [12, 13], and imaging typically shows hypoechoic lesions on ultrasonography [14, 15] and diffuse or focal low‐intensity lesions on T2WI MRI, findings that often mimic prostate cancer. Consequently, many cases are diagnosed only by biopsy [14, 16]. In our case, despite negative Ziehl–Neelsen staining, the diagnosis of prostate TB was supported by prior positive urine culture and characteristic histopathological findings, which also excluded malignancy. In the present case, mild PSA elevation and T2WI–low/DWI–high MRI nodules made it difficult to exclude prostate cancer prior to biopsy. Although prostate TB following intravesical BCG therapy has been reported [16, 17, 18], no previous cases have described concomitant bladder cancer and prostate TB at initial presentation. Notably, existing evidence indicates that a history of TB does not increase the risk of prostate cancer [2]. However, given that there have been reports of the synchronous occurrence of prostate TB and prostate cancer [19], PSA screening appears to be necessary.

In this case, untreated diabetes was identified concurrently with TB. Patients with diabetes have a threefold increased risk of developing TB, suggesting a shared high inflammatory immune response between individuals with TB and those with diabetes [20]. These findings further underscore the importance of routine health checkups for the prevention of lifestyle‐related diseases, as well as early detection and timely therapeutic intervention.

This report highlights several important clinical considerations. First, in patients with persistent sterile pyuria despite antibiotic therapy, UGTB should be considered as an important differential diagnosis. Second, individuals with pulmonary TB should be carefully evaluated for concomitant UGTB and recognized as having an increased risk for urothelial carcinoma. Regular urinalysis, urinary tract ultrasonography, and cystoscopy when necessary are essential for early detection.

## 4 Conclusion

We reported the first known case of concurrent bladder urothelial carcinoma and prostate TB. In routine clinical practice, it may be important to be aware that patients with UGTB could have an increased risk of developing urothelial carcinoma.

## Ethics Statement

The authors have nothing to report.

## Consent

Written informed consent was obtained from the patient for publication of the details of this medical case and any accompanying images.

## Conflicts of Interest

The authors declare no conflicts of interest.

## Data Availability

Data sharing not applicable to this article as no datasets were generated or analysed during the current study.
